# Low Power Consumption Hybrid-Integrated Thermo-Optic Switch with Polymer Cladding and Silica Waveguide Core

**DOI:** 10.3390/polym14235234

**Published:** 2022-12-01

**Authors:** Yuqi Xie, Jiachen Han, Tian Qin, Xuyang Ge, Xihan Wu, Lu Liu, Xubin Wu, Yunji Yi

**Affiliations:** 1 College of New Materials and New Energies, Shenzhen Technology University, Shenzhen 518118, China; 2 College of Integrated Circuits and Optoelectronic Chips, Shenzhen Technology University, Shenzhen 518118, China

**Keywords:** thermo-optic switch, hybrid-integrated, polymer cladding, air trench

## Abstract

Taking advantage of the large thermo-optical coefficient of polymer materials, a hybrid-integrated thermo-optic switch was designed and simulated. It is also compatible with the existing silica-based planar light-wave circuit (PLC) platform. To further reduce the power consumption, we introduced the air trench structure and optimized the structural parameters of the heating region. This scheme is beneficial to solving the problem of the large driving power of silica-based thermo-optic switches at this stage. Compared with the switching power of all-silica devices, the power consumption can be reduced from 116.11 mW (TE) and 114.86 mW (TM) to 5.49 mW (TE) and 5.96 mW (TM), which is close to the driving power of the reported switches adopting polymer material as the core. For the TE mode, the switch’s rise and fall times were 121 µs and 329 µs. For the TM mode, the switch times were simulated to be 118 µs (rise) and 329 µs (fall). This device can be applied to hybrid integration fields such as array switches and reconfigurable add/drop multiplexing (ROADM) technology.

## 1. Introduction

The optical switch is a crucial element of optical computing and optical networks as well as an essential component of the all-solid-state LIDARs. At this stage, the scale of the switching array is now limited by loss, power consumption, and other critical indexes. Currently, optical switches are classified as electro-optic switches or thermo-optic switches based on their operating principles. Electro-optic switches have a rapid response time, but free carriers cause additional light absorption and there is 1–2 dB loss per switch. For this reason, electro-optic switches are not conducive to large-scale array implementation. The thermo-optic switch is another large-scale array solution. On the material side, inorganic and polymer materials are the main materials for thermo-optical switches. The miniaturization requirements can be met by using silicon with a high refractive index [1,2,3]. However, devices that are too small will introduce coupling loss problems during the coupling process and increase process costs. Currently, programs such as photonic wire bonding (PWB) can be used to tackle the loss problem. Due to its low coupling loss with optical fiber (the refractive index matches the fiber), excellent stability, low transmission loss, and low cost for the lithography process, silica has emerged as the main material for current commercial passive components of optical communication. However, because of the low thermal-optic coefficient of silica, the driving power consumption of silica-based thermo-optical switches is a bottleneck in the field of large-scale integrated devices [4,5]. Mikko Harjanne et al. reported a 2 × 2 inorganic thermo-optical switch with a response time of 700 ns and a driving power consumption of 260 mW [4]. Since the thermo-optical coefficient of polymer is typically ten times greater than that of silica, all-polymer switches consume less driving power than silica-based switches [6,7,8,9]. In 2011, Al-Hetar et al. proposed an all-polymer MMI-MZI thermo-optic switch with a driving power consumption of 1.85 mW and a switching time of 0.7 ms [10]. In 2014, Xibin Wang et al. fabricated an all-polymer thermo-optic switch [8]. The switching power was 5.3 mW at 650 nm and the switch on–off time was more than 0.9 ms. The all-polymer thermo-optic switch has the advantage of reducing power consumption, but the response time is long due to the small thermal conductivity of the polymer.

Combining the advantages and drawbacks of the above two types of switches, a kind of thermo-optic switch with an organic/inorganic hybrid structure has attracted a great deal of attention in recent years. N. Keil et al. reported a hybrid polymer/silica thermo-optic vertical coupler switch with a driving power consumption of 30 mW [11]. In 2012, Yunfei Yan et al. demonstrated a 2 × 2 thermo-optic switch based on the MMI-MZI structure, and the device required 6.2 mW per π shift power with a response time of 100 µs [12]. In 2014, Lei Liang et al. fabricated a polymer/silica 2 × 2 directional coupler Mach–Zehnder interferometer (DC-MZI) thermo-optic switch with a switching power of 7.2 mW and a response time of ~100 µs [13]. Hybrid structured switches can take advantage of the complementary benefits of both materials, but at present, more hybrid structured switches use silica as the lower cladding and other different polymer materials as the core and upper cladding. It is possible to reduce the power consumption while increasing the response speed. However, polymer-based thermo-optic switches still have problems with loss in commercial applications due to the poor stability of polymers. Therefore, polymer-based switches are not only incompatible with commercially available devices but also cannot replace silica switches in commercial integrated devices at this stage.

To further reduce the power consumption of the device, a thermo-optic switch with an air trench has been proposed. In 2015, Yufen Liu et al. demonstrated a polymer/silica hybrid thermo-optical switch with an air trench structure, which can significantly improve the heating efficiency by nearly three times [14]. On the other hand, the air trench structure raises the cost of processes such as lithography, masking, and etching.

Moreover, to improve the performance of the thermo-optic switch, polymeric thermo-optic switches integrated with graphene and other materials have been studied [15,16,17]. In 2019, Xibin Wang et al. buried graphene electrodes in a polymer waveguide, reducing the switch power consumption roughly four-fold with rise and fall times of 1.58 ms and 1.57 ms [18]. In 2020, Yue Sun et al. reported a thermo-optic switch with a graphene-assisted heating layer, which consumed only 0.39 mW, with a rise time of 30 µs and a fall time of 92.4 µs [19]. Device performance can be enhanced by organic–inorganic composite materials such as graphene-doped polymer materials. However, organic–inorganic systems have large-scale film-forming difficulties, and the application of two-dimensional materials may cause additional loss, so currently these materials have not been employed commercially to solve the problem of large-scale device integration.

In recent years, nanoimprint lithography has been widely used in the fields of augmented reality (AR) and virtual reality (VR), which is a low-cost and high-precision processing scheme for polymer devices [20,21,22]. Polymer upper cladding and the air trench structure can be fabricated at a lower cost on the silica core by nanoimprinting. Therefore, in this study, a low-power thermo-optic silica-based switch with polymer upper cladding and an air trench structure was proposed. This method is also compatible with the manufacturing of silica-based PLC chips. Moreover, the design of the heating arm allowed more distribution of optical power in the polymer layer, improving the heating efficiency. Compared with the power of an all-silica optical switch, the power consumption of this device was reduced by about 95%. Compared with the polymer-based device, this device is more suitable for practical applications due to the low loss and stability of the silica core, which is also compatible with the current commercial platforms.

## 2. Design and Optimization

### 2.1. Device Structure Design

**Figure 1:**
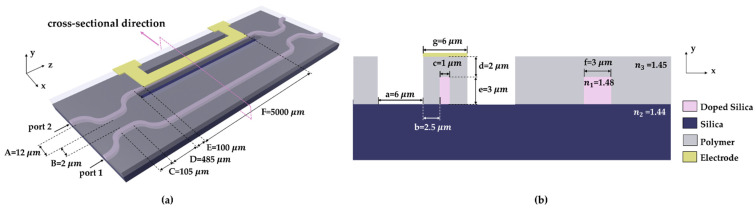
The structure of the silica/polymer hybrid thermo-optic switch in (**a**) a three-dimensional direction and (**b**) a cross-sectional direction, including heating arm, non-heating arm, air trenches, and electrode. A, B, C, D, E, and F are the offset of the bend waveguide, the waveguide spacing in the directional coupling area, the length of the straight waveguide, the length of the bend waveguide, the length of the taper waveguide, and the length of the electrode.

In terms of structure, the switch designed in this paper is divided into two 3 dB directional couplers, a phase turning area, two taper waveguides, air trenches, and a heating electrode, as shown in Figure 1a. A and D are the offset and the length of the bend waveguide, B and C are the waveguide spacing and the length of the straight waveguide in the directional coupling area, and E and F are the taper waveguide and the length of the heating area. The cross-sectional view of the phase turning area is shown in Figure 1b. The thickness of the upper cladding was d = 2 µm, which is thick enough to minimize the optical loss caused by the metal electrode. The height of the core was e = 3 µm and the width of the non-heating arm was f = 3 µm. In the heating region, the width of the core layer was c = 1 µm. The width of the air trench was a = 6 µm, and b = 2.5 µm was the distance between the air trench boundary and the core. The width of the electrode was set to be g = 6 µm. We settled on a heating zone (electrode) length of 5000 µm. The length of the non-heating zone can be used to change the operational state of the switch when the switch is not heated. For the finalized device structure, modulation arm lengths of 5020 µm and 5030 µm were selected to fulfill the switching states for the TE and TM modes when unheated. In terms of material, we selected a doped silica core layer ($n_{1}\;$= 1.48) and silica as the substrate ($n_{2}\;$= 1.44). To ensure a greater optical field distribution in the polymer upper cladding, we set the refractive index of the polymer cladding as $n_{3}$= 1.45, which is slightly higher than the refractive index of the substrate.

### 2.2. Optimization of Cross-Sectional Optical Field

**Figure 2:**
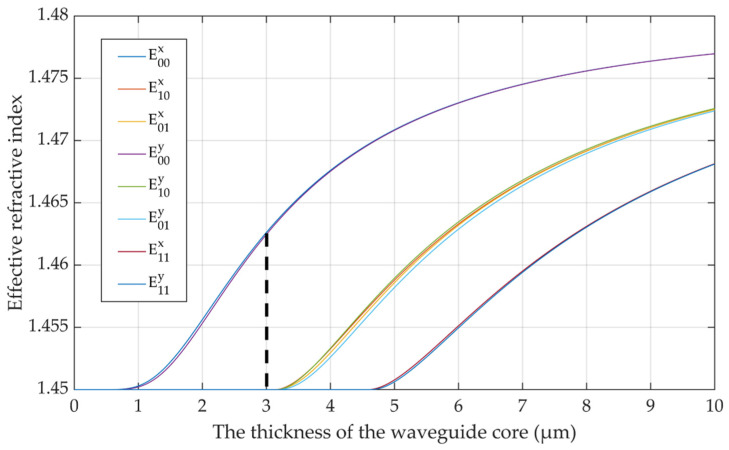
Single-mode condition curve. Dashed line indicates: 3 × 3 µm is the maximum size to ensure single-mode transmission.

We calculated the relationship between the effective refractive index of different modes and the thickness of the silica waveguide core. As shown in Figure 2, 3 × 3 µm is the maximum size to ensure single-mode transmission. Thus, the dimension of the core can be set to 2 × 2 µm or 3 × 3 µm.

**Figure 3:**
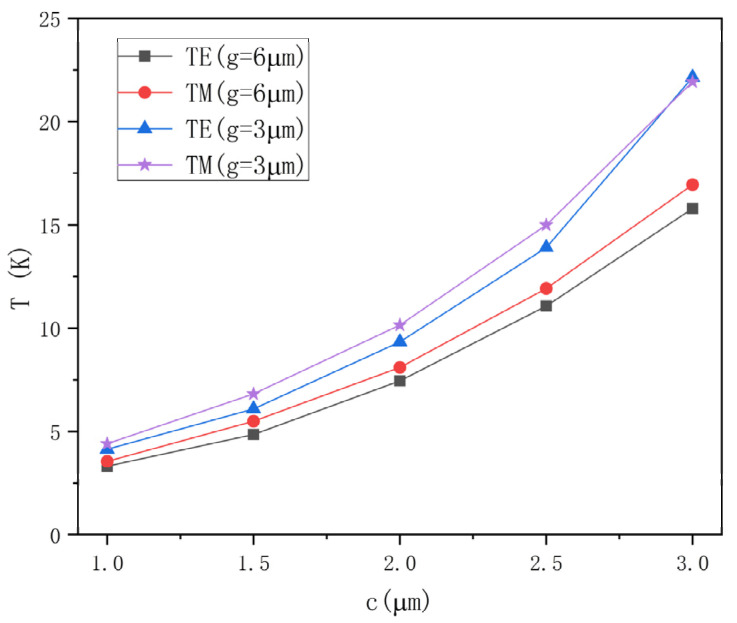
Relationship between the minimum temperature and the width of the core in the heating region for the electrode widths of 3 and 6 μm.

**Figure 4:**
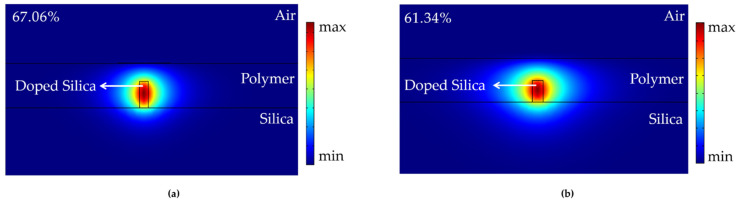
Optical field distribution of the heating arm (**a**) when the width-to-height ratio of the core is 1:3 and (**b**) when the width-to-height ratio of the core is 1:2.

**Figure 5:**
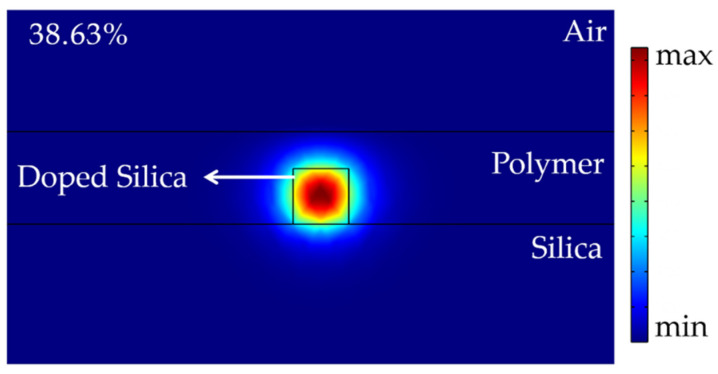
Optical field distribution of the non-heating arm.

According to the principle of thermo-optic switching, one side of the arms is heated to change the effective mode refractive index of the waveguide, resulting in a shift in phase and enabling the device to achieve the switching function. After determining the length of the heating region, the effective mode refractive index difference which is required to achieve a certain phase difference between the two arms can be calculated by Equation (1) [23]:

(1) $$\Delta \varphi =\frac{2\pi }{\lambda }\Delta NL,$$

where Δφ is the waveguide phase change, λ is the optical wavelength, ΔN is the variation of the phase difference between the two arms due to heating, and L is the length of the heating region. Due to the higher thermal-optic coefficient of the polymer, heating to the same temperature can lead to a greater change in the refractive index. Therefore, to spread more optical power throughout the polymer, the width of the core in the heating region was reduced. At the same time, the dimension of the core for the other arm remained 3 × 3 µm, forming an asymmetric structure. For this sort of asymmetric structure, the initial phase can be adjusted by changing the length of the non-heating region. Accordingly, we maintained the same length of the electrode (5000 µm) and analyzed the relationship between the width of the core and the required temperature for switching. The effective mode refractive index can be easily modified when the width of the core reduces and the horizontal limit of the optical field decreases, increasing the optical power percentage of the polymer. Figure 3 illustrates that the smaller the core width, the lower the minimum temperature to realize the switching function, whether the electrode width is 3 or 6 μm. Due to the machining accuracy of lithography, the width of the core in the heating region was set to 1 μm. The optical power percentage was 67.06% when the width-to-height ratio of the core in the heating area was 1:3 and 61.34% when it was 1:2. Therefore, to achieve a higher optical power percentage of the polymer, the height of the waveguide core was set as 3 μm. The optical field distributions were simulated, as shown in Figure 4a,b. In the non-heating area, to achieve polarization insensitivity, the width of the core was chosen to be equal to its height. Consequently, the core width and height of the waveguide in the non-heating area were both chosen as 3 μm. The optical field distribution is shown in Figure 5, and the proportion of optical power in the polymer was 38.63%.

### 2.3. Optimization of Cross-Sectional Thermal Field

**Figure 6:**
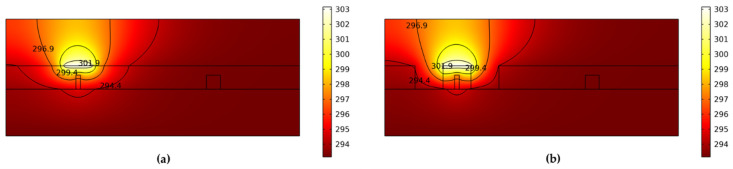
The cross-sectional thermal distribution of the device (**a**) without an air trench and (**b**) with an air trench.

To reduce the switching power, the air trench structure was introduced. When we set the thermal conductivity of the polymer to a general value (0.19 W/mK), the cross-sectional thermal distribution of the device without an air trench was as shown in Figure 6a. Figure 6b shows the result with the air trench structure. The electrode was also heated by 10 K. Since the thermal conductivity of air (about 0.023 W/mK) is much lower than that of polymer, the air trench can diminish the effective width of heat diffusion, preventing heat loss and reducing driving power.

**Figure 7:**
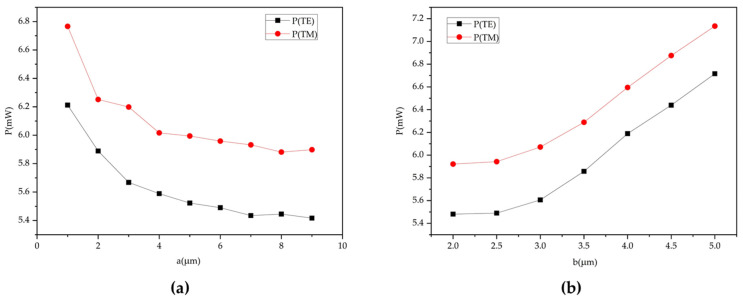
(**a**) Relationship between the switching power and the width of the air trench. (**b**) Relationship between the switching power and the distance from the air trench boundary to the core.

The width and location of the air trench need to be optimized. The air trench can improve the heating efficiency, but as the width increases, the effect of enhancing thermal field efficiency tends to be gradual. When the air trench width was greater than 6 μm, the influence of the width variation on driving power could be ignored, as illustrated in Figure 7a. Therefore, the width of the air trench was selected to be 6 μm. The heating electrode width was equal to the waveguide width. After determining the air trench width to be 6 µm, we investigated the effect of the placement of the air trench on the drive power consumption. As shown in Figure 7b, when the distance from the air trench to the core was 2 and 2.5 µm, the required power to modulate the phase shift from 0 to π was the lowest. With the purpose of decreasing the loss caused by mode mismatch, the distance was selected to be 2.5 µm.

### 2.4. Optimization of the Coupling Structure

The parameters of the directional couplers need to be optimized, and we used the beam propagation method (BPM) to scan for proper structural parameters. The final structural parameters were as follows. In the 3 dB directional couplers, the straight waveguide was 105 µm, the bend waveguide length was set at 485 µm, and the waveguide spacing in the directional coupling area was chosen to be 2 µm. The offset of the bend waveguide was 12 µm to realize a 3 dB wave splitting and combining state. The directional coupler and heating arm were connected via a taper waveguide, which had a length of 100 µm and was enough to reduce the loss.

**Figure 8:**
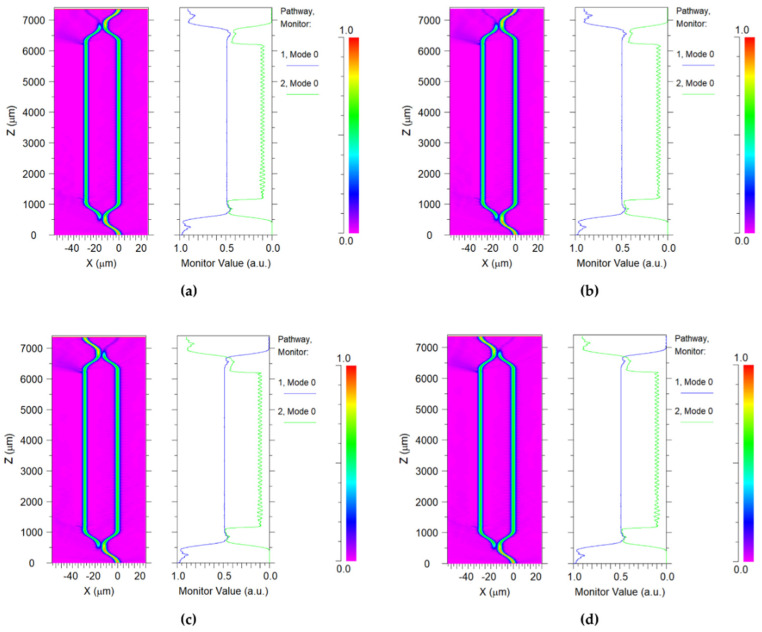
Simulated optical distribution for (**a**) TE mode and (**b**) TM mode when no driving power was applied to the heater, and for (**c**) TE mode and (**d**) TM mode when driving power was applied to the heater.

The switch function was simulated by BPM. The optical distributions in different states are shown in Figure 8. The optical power was launched in port 1. If no driving power is applied to the heater, the optical power will export from the end of port 1 and the final output energy of port 2 will be 0. This state is called the “bar state”, as shown in Figure 8a (for TE mode) and Figure 8b (for TM mode). If an appropriate voltage is applied to the electrode, the phase change between the two arms will be produced. The optical power can be output from port 2. This situation is referred to as the “cross state”, as demonstrated in Figure 8c (for TE mode) and Figure 8d (for TM mode). The transmission loss was measured at about 0.46 dB.

## 3. Device Performance Simulation

### 3.1. Power Consumption

**Figure 9:**
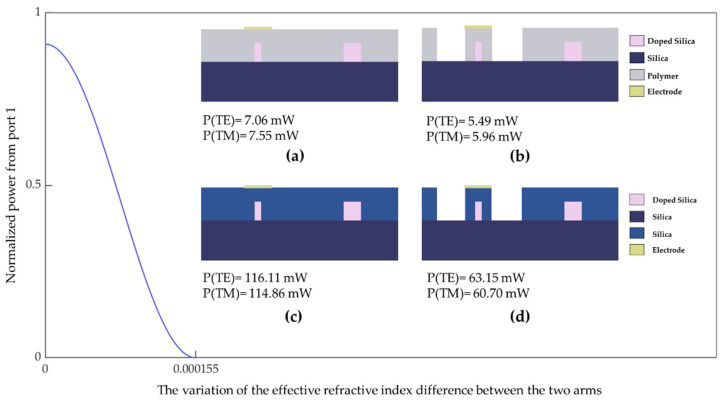
The power consumption to realize the switching function for: (**a**) a polymer/silica device without an air trench, (**b**) a polymer/silica device with an air trench, (**c**) an all-silica device without an air trench, and (**d**) an all-silica device with an air trench.

The driving power consumption adopting different structures (device with or without an air trench) and variable materials (upper cladding using silica or polymer) was calculated by the finite element method (FEM). The relationships between the switching power and different devices are shown in Figure 9. To make the shift of the phase difference achieve π, the all-silica thermo-optic switch without an air trench needed 116.11 mW for TE mode and 114.86 mW for TM mode. The all-silica device with an air trench achieved its intended switching function and the calculated power consumption was 63.15 mW (TE) and 60.70 mW (TM). Compared with the all-silica switch, the polymer/silica switch demands a lower driving power. For the polymer/silica switch without an air trench, the applied electric power as the switching power was 7.06 mW (TE) and 7.55 mW (TM). The polymer/silica device with an air trench demanded 5.49 mW (TE) and 5.96 mW (TM). Hence, devices with an air trench have the advantage of low power consumption. In addition, the polymer upper cladding is beneficial in reducing power consumption because the thermal-optic coefficient of polymer (−2 × 10^−4^ K^−1^) is typically ten times that of silica (2 × 10^−5^ K^−1^). In summary, the hybrid-integrated structure with an air trench can effectively reduce the driving power for silica-based switches. In this study, through adjusting the distribution of the optical field in the polymer material and optimizing the parameters of the heating region, power consumption was reduced by about 95% versus the switching power of an all-silica device.

### 3.2. Switching Time

**Figure 10:**
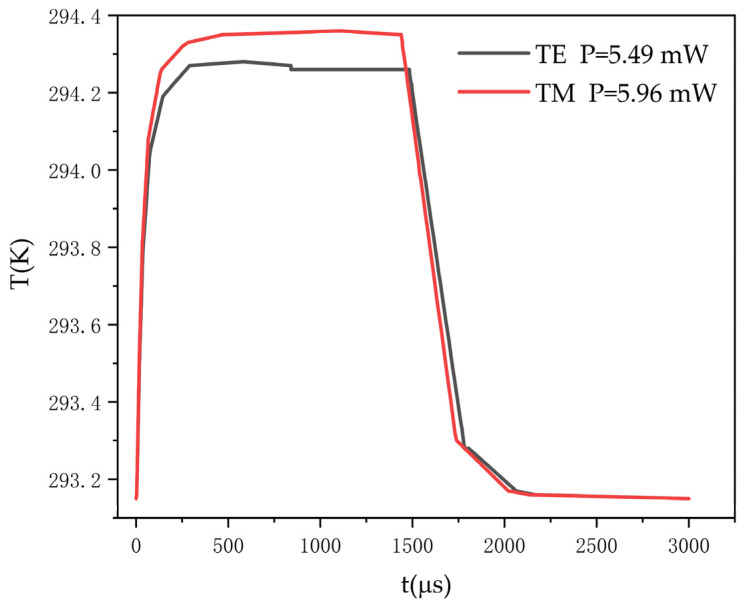
The response times for the polymer/silica switch with an air trench for TE mode and TM mode.

When calculating the switching time, we focused on heat conduction versus time, so transient thermal analysis was chosen for the calculation. The transient temperature change was obtained using the heat conduction equation [24]:

(2) $$\rho c_{P}\frac{\partial T}{\partial t}=k\nabla^{2}T+Q\left(x,y,z,t\right),\;$$

where ρ is the density of the material, $c_{P}$ is the specific heat capacity, k is the thermal conductivity of the material, and $Q\left(x,y,z,t\right)$ is the heat generation rate per unit volume. The rise and fall times of the polymer/silica switch with an air trench were simulated, as shown in Figure 10.

### 3.3. Effect of Polymer Thermal Conductivity

According to the equation used to compute the power consumption, it makes sense to infer that power consumption is proportional to thermal conductivity [25]. With the same thermo-optic coefficient, the polymer cladding material with lower thermal conductivity has the advantage of reducing power consumption. However, the switch response time may be correspondingly increased. Conversely, if more emphasis is placed on the switching time, polymer materials with high thermal conductivity can be employed. The application of polymers with high thermal conductivity can bring a significant improvement in response speed at the cost of higher power consumption. Consequently, various polymers can be selected in practical applications based on different requirements.

**Figure 11:**
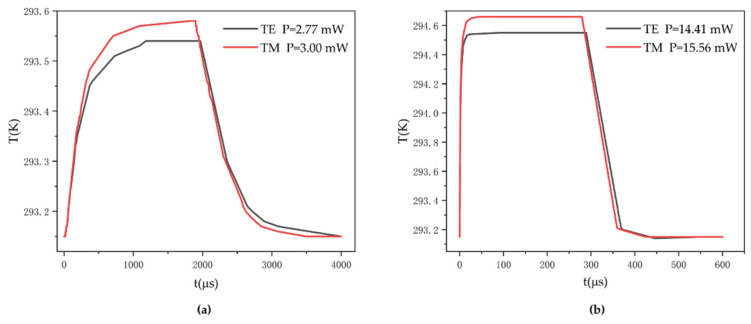
Simulation of the relationship between temperature and the time of the devices (**a**) using low thermal conductivity polymer (0.0125 W/mK) and (**b**) using high thermal conductivity polymer (16 W/mK).

Polymer materials can achieve low or high thermal conductivity by using modification techniques. Based on published reports, the thermal conductivity of the polymer can be reduced to 0.0125 W/mK [26]. At the same time, studies on polymers with high heat conductivity have also made some progress [27,28,29]. A kind of polymer with high thermal conductivity (about 16 W/mK) has been reported [27]. The influence of polymer materials with special physical properties on the thermo-optic switch indexes was analyzed, as shown in Figure 11. For polymer materials with low thermal conductivity, we selected a value of 0.0125 W/mK for analysis. The device required a heating power as low as 2.77 mW for TE mode and 3.00 mW for TM mode. Nevertheless, the response times both exceeded 1.3 ms, which were much longer than that of the device using polymer cladding with general thermal conductivity (0.19 W/mK). For a high thermal conductivity, the value was set to 16 W/mK to simulate. The device required 14.41 mW and 15.56 mW per π shift power for TE mode and TM mode. However, the rise times were reduced to 6.0 µs and 6.5 µs, and the fall times were decreased to 121 µs and 123 µs. The response speed remarkably improved.

## 4. Discussion and Conclusions

The results of the thermal optical switch in this paper are also compared with those of other reported thermal optical switches based on other waveguide structures, as shown in Table 1. Polymer cladding and the air trench can reduce the power consumption of this silica-based thermo-optic switch. We can see that the power consumption of the switch that we proposed in this paper was the lowest versus other thermal optical switches with inorganic material as the waveguide core. At the same time, the switching power was also close to that of most polymer-based switches reported at this stage. The polymer cladding also allowed the device to achieve loss compensation or all-optical control by later doping in the polymer cladding. The use of polymer cladding not only reduced the power consumption of the switch but also provided more possibilities and potential prospects for the application of this device.

In this article, a silica/polymer hybrid thermo-optic switch that is compatible with the silica PLC chip was proposed. In terms of the waveguide structure, the optimization of the waveguide structure (the width-to-height ratio of the core was 1:3) improved the optical power proportion in the heating area by around 1.7 times. In terms of the device structure, by introducing taper, an air trench, and polymer upper cladding, the power consumption of the silica thermo-optic device was significantly reduced. At the wavelength of 1550 nm, the power consumption was 5.49 mW and 5.96 mW, which was approximately 95% of power saved compared with that of an all-silica switch. For TE mode, the rise and fall times were 121 μs and 329 μs. For TM mode, the rise and fall times were 118 µs and 329 µs. To compare with the reported devices, the device performance when the length of the electrode is 1 mm was also calculated. The power consumptions for TE and TM modes were 5.135 mW and 5.828 mW. The switch responded in around 430 µs. This switch showed a low power consumption and fast response speed in comparison to the reported switches. In terms of material, we also discussed the effect of special polymer thermal conductivity on the thermo-optic switching performance. The results showed that when the polymer with a lower thermal conductivity was selected, the switching power consumption could be reduced (by about 50%), but the switching response time may be longer. On the contrary, the device using a higher thermal conductivity polymer could provide a faster response speed at the expense of increased power consumption.

## Figures and Tables

**Table 1:** Comparisons among the performances of this thermal optical switch and those of other reported thermal optical switches.

Reference	Structure (Core/Upper Cladding/Under Cladding)	Wavelength (nm)	PC (mW) ^1^	RT (µs) ^2^	FT (µs) ^3^
[5]	Silicon/Silica/Silica	1520–1630	40	30 (total)	
[4]	Silicon/Silica/Silica	1550	260	0.725	0.700
[8]	Doped PMMA/PMMA/PMMA	650	5.3	464.4	448
[30]	EpoCore/EpoClad/EpoClad	850	4.5	400	600
[13]	SU-8/PMMA/Silica	1550	7.8	100 (total)	
[31]	SU-8/PMMA/Silica	1550	7.2	106	93
[14]	SU-8/PMMA/Silica	1550	3.4	183.1	139.9
This work	Doped Silica/Polymer/Silica	1550	5.49/5.96	121/118	329/329

^1^ PC, power consumption; ^2^ RT, rise time; ^3^ FT, fall time.

## Data Availability

The data that support the findings of this study are available from the corresponding author upon reasonable request.
